# Psychometric properties of the BALCI Turkish version

**DOI:** 10.1186/s41155-023-00248-0

**Published:** 2023-02-28

**Authors:** Oğuz Mercan, Fedai Kabadayı

**Affiliations:** 1 Ministry of Education, Eskişehir, Türkiye; 2 Graduate School of Educational Sciences, Gazi University, Ankara, Türkiye; 3 Department of Counseling and Guidance, Recep Tayyip Erdogan University, Rize, Türkiye

**Keywords:** Beliefs about losing control inventory, Obsessive–compulsive disorder, Adaptation, Confirmatory factor analysis, Emerging adults

## Abstract

The present study aimed to adapt and validate the Beliefs About Losing Control Inventory (BALCI) in the non-clinical Turkish emerging adults sample. The study group consisted of 549 participants from three study phases and aged between 18 and 28 years and mainly women. According to the results of the confirmatory factor analysis (CFA), the BALCI Turkish Version (BALCI-TV) confirmed 21 items in three factors. The network analysis findings showed that the items including the factors were together. The results indicated that configural, metric, scalar, and strict invariance across the gender. Cronbach’s α and McDonald’s ω of the total BALCI-TV were .90. Test–retest correlation result was .89. All results indicated that the BALCI-TV had good psychometric properties. The BALCI-TV can be provided to measure control and beliefs related to control within obsessive–compulsive disorder for Turkish academics and mental health practitioners.

## Introduction

Obsessive–compulsive disorder (OCD) is defined as a psychiatric disorder that is expressed as repetitive behavior or recurrent and persistent thoughts that are sometimes forced and unintentional and cause anxiety or distress (APA, 2013). These thoughts emerge in structures such as contamination, cleaning, sexuality, harm, and control (Abramowitz et al., 2008) and OCD is associated with compromising quality of life (Franklin & Foa, 2011). Simply, obsessions are defined as uncontrollable thoughts, impulses, and images that exacerbate anxiety. On the other hand, compulsions are repetitive behaviors or cognitive actions that occur as a reaction and describe tasks surrounded by harsh rules to reduce the distress caused by obsessions (Richter & Ramos, 2018). Loss of control has a key point in the theoretical background of OCD (Carr, 1974; Gillan et al., 2016; McFall & Wollersheim, 1979). Beliefs that losing control is hazardous are emphasized in cognitive models of OCD (Gagné & Radomsky, 2017). Specific areas of dysfunctional belief associated with OCD include excessive responsibility and perfectionism, as well as the need to control thoughts (Julien et al. 2008). Therefore, control and loss of control among these structures have recently come to the fore in the context of cognitive therapy (Radomsky & Gagné, 2020). The control expressed here refers to controlling intrusive thoughts (Amir et al., 1997).

Many people have annoying thoughts, and many thoughts seem to come to our minds throughout the day, and many of these thoughts are inherently beyond our control. The effort to control these thoughts is quite natural, but it is not possible to fully take control and such a strategy is likely to fail (Purdon & Clark, 2002). If people start losing or doubting their sense of control over their environment and themselves, then intense anxiety may follow (Clark, 2004). However, the fear of losing control due to unsuccessful control efforts will increase with it (Gagné & Radomsky, 2020). Thus, as one has high beliefs about the state of losing control, anxiety also increases significantly (Kelly-Turner & Radomsky, 2020). Radomsky et al. (2008) state that it is more realistic to think about the behavior of control in two categories normal controlling and pathological controlling rather than grading on a dimension that is healthy on one end and sick on the other. Attempts to control thought mentally result in failure causing the individual to behave compulsively. Although these behaviors might at first provide short-term relief, efforts at long-term control is detrimental to the individual (Clark, 2004). Unlike healthy people, obsessives have a clear and widespread need for control in all areas of their lives. “Control” appears to be an object rather than a tool for environmental compliance (Mallinger, 1984). However, it is emphasized that the concept of control in the context of OCD should be evaluated whether it is problematic over time rather than environmental or objective (Radomsky et al., 2014).

Compared to other psychiatric disorders, OCD patients are known to have wider control repertoires (Ladouceur et al., 2000; Rosa-Alcázar et al., 2021). Suppressing thoughts and diverting attention are the main behaviors that are considered thought control efforts (Williams & Wetterneck, 2019). Despite all these efforts, in some studies, people with OCD have failed the control of thoughts, and, accordingly, feared a loss of control over their thoughts, behaviors, emotions, body, and/or bodily functions (Clark & Purdon, 1993; Reuven-Magril et al., 2008). This fear can be considered with the emergence of maladaptive beliefs in OCD, which is included in cognitive theory (Gagné & Radomsky, 2017). The stated irrational thoughts appear as excessive preoccupation with monitoring their thoughts and actions, applying strict rules in guiding their behaviors, and taking heavy restrictions to avoid losing control in people diagnosed with OCD (Reuven et al. 2008). In this sense, we say that there is a positive correlation between OCD symptoms and fear of losing control (Froreich et al., 2016) and control of thoughts (Aardema et al., 2021). Similarly, all these notes that various obsessions of individuals within obsessions are gathered around the fear of losing control (Rachman, 2007). In the case of control, OCD has a critical place among cognitive approaches, especially in areas such as responsibility and controlling thoughts (Whittal et al., 2010). In this sense, studies have shown that beliefs about the control of thoughts are a predictor of OCD symptoms (Myers et al., 2008) and emphasize that it is important for OCD to determine strategies for thought control (Amir et al., 1997). For these reasons, we believe that there is a need for measurement instruments to determine control beliefs in both the OCD and non-OCD population.

There are some scales related to OCD and OCD-related disorders in Turkish (Besiroglu et al., 2005; Boysan et al., 2010, 2015, 2016; Guler et al., 2016; Inozu & Yorulmaz, 2013; Safak et al., 2018; Secer, 2014; Trak & Inozu, 2017; Yorulmaz & Gencoz, 2008; Yorulmaz et al., 2015, 2019; Yucelen et al., 2006). Some of these measurement instruments are known to have an emphasis on control. In this sense, controlling thoughts (Boysan et al., 2010; Yorulmaz & Gencoz, 2008), doubt-control (Secer, 2014), impaired control over mental actions and impulses, and concerns about loss of control over behavior (Besiroglu et al., 2005) constitute the structures dealing with control in measurement instruments. According to these studies, the structures dealing with control in OCD are mostly related to the control of thoughts. Maladaptive beliefs play an important role, especially in OCD (Radomsky & Gagné, 2020). A dimension related to losing control remains limited including OCD-related behavior, emotions, the importance of staying in control, and body/bodily functions. Beliefs About Losing Control Inventory (BALCI) provides a comprehensive perspective on beliefs about losing control. The first dimension deals with “thoughts, behavior, and emotions” and focuses on the control of “thought, behavior, and emotions. The second dimension is related to “the importance of staying in control” and focuses on maintaining control. Finally, “beliefs about losing control over one's body/bodily functions” are associated with psychosomatic symptoms. In other words, BALCI presents a unique structure different from previous measurement instruments. We believe that this structure will provide a considerable measure for Turkish culture. Thus, BALCI Turkish Version (BALCI-TV) will fill an important contribution to Turkish literature.

The present study aimed to adapt and validate the Beliefs About Losing Control Inventory (BALCI) in the non-clinical Turkish emerging adult population. Considering that contamination and cleaning have a critical bond in control (Gelfand & Radomsky, 2013) and assuming that uncertainty occurs in an uncontrolled area this aspect of the research also constitutes a different structure. On the other hand, considering that individuals lose their control over more than one psychological function (Gagné, 2021), we believe that it is important for BALCI to focus on emotion-thought-behavior, and bodily functions. Hereby, this inventory aims to evaluate a person’s degree of fear of losing control, the meaning and perceived negative results of losing control, and beliefs in the importance of staying in control (Radomsky & Gagné, 2020).

## Method

### Participants

The BALCI correlations between the original and Turkish version study included 31 bilingual participants (study 1). There were 436 participants for studies including construct validity, network analysis, and test-criterion and reliability (study 2). The sample consisted of 436 (328 women, 108 men) Turkish emerging adults. Participants were studying at different universities. The mean age of the participants was 21.82 (SD = 1.98, range = 18—28). Finally, there were 82 participants in the test–retest study (study 3).

### Instruments

We used BALCI, Obsessive–Compulsive Inventory-Revised, and Obsessive Beliefs Questionnaire in the present study.

#### Beliefs About Losing Control Inventory (BALCI)

The original version of the BALCI was developed by Radomsky and Gagné (2020). The BALCI measures a self-report of negative beliefs about losing control. The scale consists of 21 items and three dimensions. The sub-dimensions are thoughts, behavior, and emotions (factor 1), beliefs about the importance of staying in control (factor 2), and beliefs about losing control over one's body/bodily functions (factor 3). The scale is a 5-point Likert type (0 = not at all, 4 = very much). The sample item in the first factor for BALCI is “I'm afraid that I might not be able to keep my emotions in check”, “It's important for me to stay in control of my thoughts” for the second factor, and “I am afraid of losing control of my bladder and/or bowels” for the third factor. Cronbach’s alpha (α) of the total BALCI was 0.93 in the sample. Item loads of the scale are minimum 0.40 for the sample (Radomsky & Gagné, 2020).

Several procedures were applied for the translation and back-translation of the study into Turkish for Beliefs About Losing Control Inventory (International Test Commission, 2017). First, five bilingual specialists translated BALCI into Turkish. Then, professional opinion was received for the BALCI**-**TV from five academicians who are experts in their profession (Counseling and Guidance, Clinical Psychology, and Cognitive-Behavior Therapy). The BALCI-TV was obtained with the last adjustments made by the authors. Then, three specialists back-translated the BALCI-TV to English. Finally, the translated form of BALCI with the original BALCI was compared.

#### Obsessive–Compulsive Inventory-Revised (OCI-R)

The original version of the OCI**-**R was developed (Foa et al., 2002). The Turkish version of the OCI**-**R was adapted (Yorulmaz et al., 2015). The scale consists of 18 items and 6 sub-dimensions. The scale evaluated distress associated with the indications of obsessive–compulsive disorder. The scale is a 5-point Likert type (0 = not at all, 4 = extremely). Cronbach’s alpha (α) values are between 0.73 and 0.90 (Yorulmaz et al., 2015). Cronbach’s alpha (α) and McDonald’s omega (ω) of the total OCI-R was 0.90 in the present study.

#### Obsessive Beliefs Questionnaire (OBQ-9)

OBQ**-**9 was designed by Obsessive Compulsive Cognitions Working Group to evaluate OCD**-**specific beliefs (OCCWG, 2001). The scale is a 7-Likert type scoring (1 = strongly disagree, 7 = strongly agree). The Turkish version of the OBQ**-**9 was adapted (Yorulmaz et al., 2019). The scale of short form consists of nine items and three dimensions. The scale is a 7-point Likert type (1 = strongly disagree, 7 = strongly agree). Cronbach’s alpha (α) values are between 0.70 and 0.75. Item loads of the scale are between 0.47 and 0.83. (Yorulmaz et al., 2019). Cronbach’s alpha (α) of the total OBQ-9 was 0.75 and McDonald’s Omega (ω) of the total OBQ-9 was 0.76 in the present study.

### Procedure

Firstly, permission was obtained from Adam Radomsky for the Turkish adaptation of the BALCI. After that, researchers obtained research ethics approval from the Research Ethics Committee. Researchers shared all questionnaires with the university students via e-mail. In addition, the research and questionnaire form were announced to the students online. Firstly, information about the research was given and a page where identity information would be kept confidential was included. They were then asked to declare that they would voluntarily participate in this research. Participants filled out the questionnaires online using Google Forms. Participants declared that their participation in the research was voluntary. There was no missing value in the data set.

### Data analysis and criteria

None of the data in this study contain missing data. SPSS, LISREL, and Jeffrey’s Amazing Statistics Program-JASP (Goss-Sampson, 2022) were used to analyze the data. The sample size for factor analysis of measurement instruments development and adaptation studies is controversial. However, recent studies show that 300 data is sufficient as a general opinion (Worthington & Whittaker, 2006), and more than 400 in recent studies (Goretzko et al., 2021). Criteria for variables are between + 1 and − 1 for skewness, and kurtosis values were used for the normality (Buyukozturk, Cokluk, and Koklu, 2015). Cronbach’s alpha (α) (Cronbach, 1951) and recently proposed McDonald’s omega (ω) coefficients (Hayes & Coutts, 2020) of each scale were calculated. CFA was applied to adapt the scale to Turkish culture and checked the network analysis of BALCI-TV. The criteria of model fit as CFI and TLI ≥ 0.95, AGFI ≥ 0.90, RMSEA between 0.05 and 0.08, and SRMR between 0.05 and 0.10 (Bentler, 1980; Browne & Cudeck, 1993; Schermelleh-Engel & Moosbrugger, 2003). Also, we assessed to network analysis using JASP. Network analysis provide the user to analyze the network structure of variables. Using network analysis, we want to provide supporting evidence for CFA findings. Thus, we would like to present additional information about the factor structure of BALCI-TV by showing how the items is in the network structure. We performed multi-group CFA to assess potential differences across gender. We followed the measurement invariance in the direction of configural, metric, scalar, and strict, respectively. Researchers cannot choose a clear time interval for test–retest reliability. Generally, a range from 1-h to 1 year is discussed (Streiner, Norman and Cairney, 2015). In the present study, we determined a 2-week interval based on the recommendation. Medium or strong correlation scores are expected for criterion validity and test–retest reliability.

## Findings

### Reliability

We calculated the Cronbach’s alpha (α) and McDonald’s omega (ω) values using JASP. Cronbach’s alpha (α) and McDonald’s omega (ω) were 0.90 for the BALCI-TV (see Table 1).

**Table 1:** Item Analysis and Reliability

	Mean	*Sd*	Item-total correlations	**If item dropped**	
				**Cronbach's α**	**McDonald's ω**
Item 1	1.96	1.15	.49*	.90	.90
Item 2	1.54	1.08	.60*	.89	.90
Item 3	2.37	1.09	.52*	.89	.90
Item 4	1.81	1.36	.64*	.89	.89
Item 5	1.79	1.22	.53*	.89	.90
Item 6	1.61	1.43	.43*	.90	.90
Item 7	0.64	0.96	.35*	.90	.90
Item 8	1.92	1.25	.68*	.89	.89
Item 9	1.52	1.10	.59*	.89	.90
Item 10	2.28	1.31	.51*	.89	.90
Item 11	1.86	1.10	.60*	.89	.90
Item 12	1.68	1.16	.66*	.89	.89
Item 13	1.21	1.10	.60*	.89	.90
Item 14	3.23	0.88	.28*	.90	.90
Item 15	3.08	0.97	.31*	.90	.90
Item 16	2.03	1.22	.70*	.89	.89
Item 17	1.56	1.12	.61*	.89	.90
Item 18	1.74	1.09	.54*	.89	.90
Item 19	2.75	1.00	.37*	.90	.90
Item 20	0.54	1.00	.30*	.90	.90
Item 21	2.22	1.34	.50*	.90	.90
**BALCI-TV**	**1.87**	**1.14**	–	**.90**	**.90**

^*^p < .01

### Correlations between BALCI and BALCI-TV

We analyzed the correlation between the BALCI and BALCI**-**TV. In the study, 31 bilingual English teachers or translators answered by 2 weeks apart. The correlation between the BALCI and BALCI**-**TV was analyzed, and the results indicated that positive correlation (see Table 2).

**Table 2:** Correlation and descriptive statistics between English and Turkish versions of BALCI

Measures	Mean	SD	Range	Skewness	Kurtosis	Pearson Correlation
BALCI	35.32	13.78	13–72	.43	.16	.74*
BALCI–TV	33.23	13.76	2–58	–.47	-.02	

*Note*. * *p* < .001, N = 31

### Confirmatory factor analyses of the BALCI-TV

CFA was performed with LISREL 8.8 software to evaluate the model fit of the BALCI-TV. A three-factor model of BALCI-TV was tested with the 21 items. The results are as follows: *χ*^2^ = 631.84, χ^2^/df = 3.41, CFI = 0.95, TLI = 0.95, RMSEA = 0.07, and SRMR = 0.06. The χ^2^/df ratio can be used as a criterion. A valid fit was confirmed on the 21-item BALCI-TV with three factors. All items had factor loadings of 0.34 or higher (see Table 3).

**Table 3:** Factor Loads of the BALCI–TV

Items	Factor 1	Factor 2	Factor 3
Item 1	.54		
Item 2	.67		
Item 3	.57		
Item 4	.63		
Item 5	.57		
Item 8	.66		
Item 9	.65		
Item 10	.53		
Item 11	.67		
Item 12	.72		
Item 13	.66		
Item 16	.74		
Item 17	.67		
Item 18	.59		
Item 14		.84	
Item 15		.78	
Item 19		.56	
Item 6			.67
Item 7			.45
Item 20			.34
Item 21			.65

### Network analysis of structure of the BALCI-TV

**Fig. 1:**
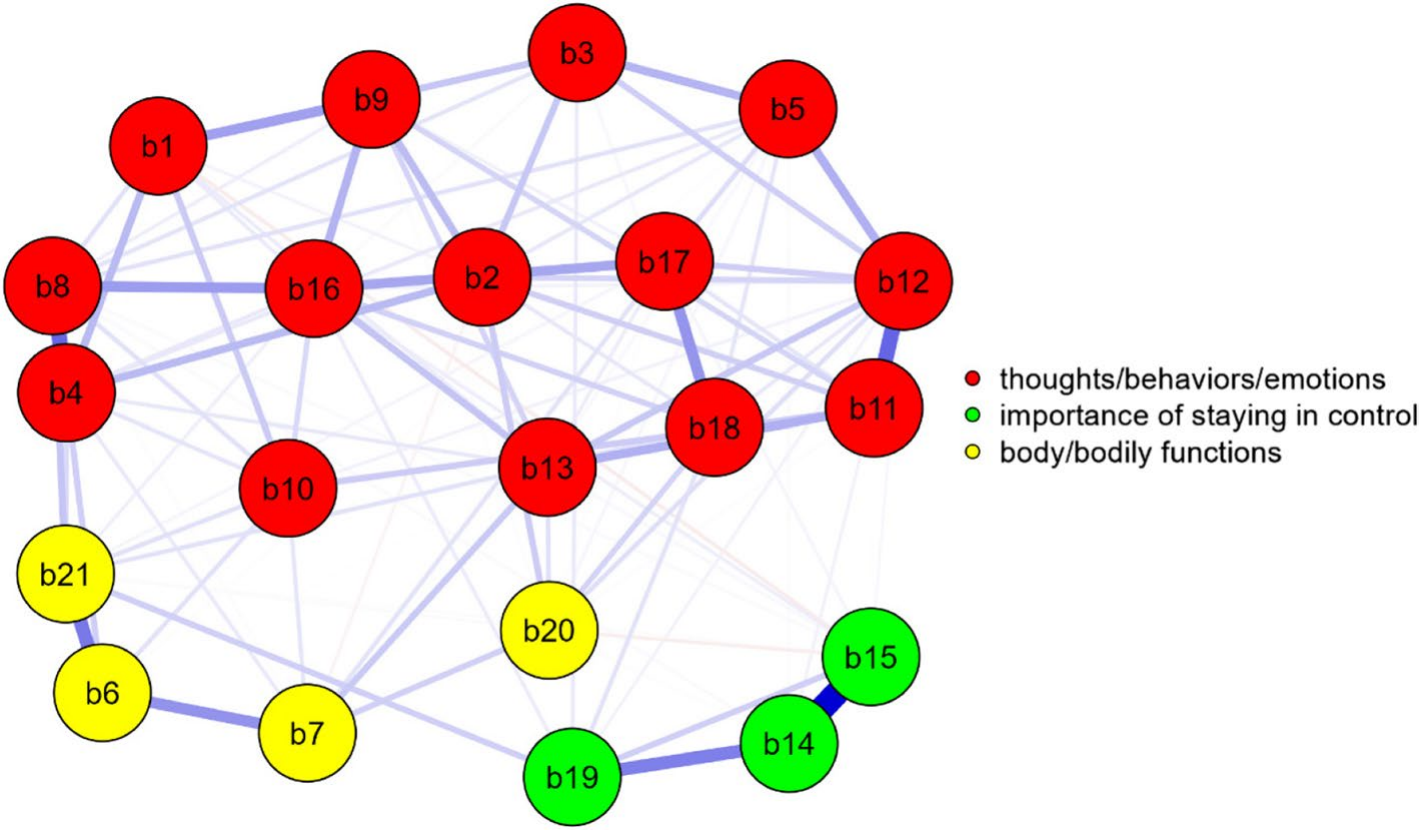
Network Analysis of the BALCI–TV

In the present study, we benefited from network analysis using JASP (Version 0.16.3). Network analysis provides information about the network structure of the variables. In networks, observed variables are referred to as nodes and estimated relations are called edges (Love et al., 2019). There were 21 nodes for 21-item BALCI-TV. Out of the 210 possible edges, the EBICglasso estimator indicated that 109 of them are non-zero, which is about 48.1%. The network structure obtained by network analysis is shown in Fig. 1.

In network structures, each item was in a circle and the lines between the circles represent the edges. In short, edges were partial correlations between two items, given all the items in the network. If the green edges indicated positive connotations, and the red edges indicated negative associations. The width and saturation of the edges indicated the absolute strength of the relationship. The position of the nodes was based on the Fruchterman Reingold algorithm, which tries to find the nodes with strong edges closer together (Wagenmakers & Kucharský, 2020). BALCI-TV includes 3 dimensions as in the original BALCI (Radomsky & Gagné, 2020). The factor structure of the measurement instrument, and each factor was presented in different colors. First, items in each dimension are related and coexist in the network plane. The red-colored items form the thoughts/behaviors/emotions dimension, the yellow-colored items form the importance of staying in control dimension, and the green-colored items form the body/bodily functions dimension on the network plane as a whole. In summary, the network analysis findings showed that BALCI-TV, which consists of 3 dimensions, essentially supports the fit indices in confirmatory factor analysis.

### Measurement invariance

We performed multi-group CFA to assess potential differences across gender (see Table 4). None of the ΔRMSEA values are greater than 0.01.

**Table 4:** Measurement Invariance of the BALCI–TV across genders

**Measurement invariance**	**χ**^**2**^		**df**	**Δχ**^**2**^	**RMSEA**	**ΔRMSEA**
Male (*n* = 108)	424.590	***	186		.010	
Female (*n* = 328)	591.737	***	186		.008	
Configural	1016.327	***	372		.089	
Metric	1034.365	***	390	18.038	.087	-.002
Scalar	1063.203	***	408	28.838	.086	-.001
Strict	1123.016	***	429	59.813	.086	.000

^***^p < .001

### Test-criterion validity

The relations among the variables BALCI-TV, OCI-R (obsessive–compulsive disorder), and OBQ-9 (obsessive beliefs) were analyzed using Pearson correlations. BALCI-TV was found to be positively correlated with obsessive–compulsive disorder (*r* = 0.57, *p* < 0.01) and obsessive beliefs (*r* = 0.47, *p* < 0.01) (see Table 5).

**Table 5:** Descriptive Statistics and Correlations

Measures	Mean	Sd	Range	Skewness	Kurtosis	1	2	3
BALCI–TV	39.34	13.88	6–82	.15	-.37	-		
OCI–R	30.77	13.52	2–72	.19	-.29	.57*	-	
OBQ–9	36.02	8.87	10–63	.16	.02	.47*	.57*	-

*Note*. *p < .01, N = 436, Cronbach’s alpha (α) for OCI–R = .90, for OBQ–9 = .75

### Reliability

Cronbach’s alpha (α) and McDonald’s omega (ω) scores were calculated for BALCI-TV’s reliability scores using JASP. Cronbach’s alpha (α) [95% confidence interval (0.90 and 0.91)] and McDonald’s omega (ω) [95% confidence interval (0.89 and 0.92)] of the total BALCI-TV was 0.90 for the CFA sample in the present study.

### Test–retest reliability

BALCI-TV was answered by 82 participants at 2-week intervals. The mean score of the BALCI-TV was 38.48 (SD = 14.11, range = 10–73) in the first study and 38.54 (SD = 14.61, range = 5–76) in the second study. Statistics on the test–retest reliability were determined using Pearson correlation. There was strong correlation between two measurements (*r* = 0.89, *p* < 0.01) (Murphy & Davidshofer, 1988).

## Discussion

The present study aimed to provide reliability and validity of the BALCI in Turkish emerging adults. Specifically, BALCI measures OCD-related beliefs about losing control. We followed several procedures systematically in the present study. Firstly, we followed the procedure of translating the scale into Turkish and then back-translating it to English. Then, CFA, network analysis, test–retest validity, and test-criterion validity were examined in the present study. Overall, the findings of the BALCI-TV in the present study were consistent with the original BALCI (Radomsky & Gagné, 2020).

As we expected, there was a strong positive correlation between the original version of BALCI and BALCI-TV. Secondly, we evaluated the factor structure of the BALCI-TV by CFA using LISREL. The BALCI-TV has good fit indices, and it confirmed 21 items within 3 factors in Turkish emerging adults according to the results of the CFA. In other words, the fit indices provided evidence that the BALCI-TV factor structure was confirmed. Factor loads ranged from 0.34 to 0.84. The phrase “If I lost control, I would throw up.” had a lower factor load (0.34) than other items of BALCI-TV. There may be two reasons for the low factor load. First, there is no vomiting in a non-clinical population. For individuals, the fear of losing control is related to the need to make sure they will not throw up (Veale, 2009). Instead, it may show other symptoms. Second, non-clinical emerging adults may experience less severe symptoms than the clinical population, and therefore the participants responded lower to this item. However, there may still be a sufficient factor load for Item-20. Furthermore, all factor loadings had a significant value accepted by the researchers. Carpenter (2018) suggested 0.32 may be a minimum score for factor loads, but any value between 0.30 and 0.40 may also be a criterion for factor loads. In addition, all the items in the present study are in the range of item factor loads recommended in the literature. Our findings supported the construct validity of BALCI-TV.

Thirdly, we wanted to provide additional evidence by indicating the tested construct of BALCI-TV on a network plane with the confirmatory factor structure using network analysis. Network analysis provides information about the relationships among the items and the direction of the relationships. In addition, the network analysis also allows us to understand the strength of the relationships between the items. The findings showed that the items had a similar structure as in the original scale. In addition, this study shows that it takes place in accordance with the structure obtained by CFA. Items in each sub-dimension were closely related to each other. At the same time, the items were positively correlated with each other, as expected. These results provide further evidence for CFA by indicating a strong aspect of the factor structure for BALCI-TV.

Fourthly Cronbach’s alpha (α) and McDonald’s omega coefficients were reported as reliability scores in the present study. The BALCI-TV reliability Cronbach’s alpha (α) and McDonald’s omega (ω) values were satisfactory. Studies in the literature consider reliability coefficient scores of 0.70 and above acceptable. Therefore, the reliability coefficients reported in the present study are satisfactory. In addition, we calculated the measurement invariance of the scale in terms of male and female participants. None of the ΔRMSEA variations was greater than 0.01 in the analyzes, indicating that BALCI-TV was invariant between men and women participants. These psychometric findings showed that the BALCI-TV was a reliable instrument.

Fifthly, The BALCI-TV has moderate correlations with OCI-R and OBQ-9 in criterion validity. Criterion validity explores the relationship between the scores obtained from the scale and the specified criteria to determine the effectiveness of the scale (Thorndike et al., 1991). In the original study, BALCI and OCD symptoms were analyzed (Radomsky & Gagné, 2020). In this sense, we believe that the current finding of the relationship of BALCI-TV with OCD symptoms as criterion validity is an important result. Considering that BALCI has a strong relationship with obsessive beliefs, anxiety, and stress (Radomsky & Gagné, 2020), we believe that the BALCI-TV is essential to design studies dealing with its relationship with obsessive beliefs, anxiety, and stress in following studies. Also, in future studies, Turkish researchers may explore the relationship of BALCI with obsessive beliefs, stress, and anxiety.

Sixthly, a strong correlation was obtained in BALCI-TV during test–retest reliability. The reliability of the BALCI-TV can be explored by using internal consistency tests, repetition with different samples, and test–retest with the same sample (Hendrickson et al., 1993). In this sense, we say that performing test–retest analysis for BALCI-TV is a considerable finding in ensuring the reliability of the scale.

Lastly, BALCI-TV will be a benchmark measurement instrument for the following studies to be conducted in the Turkish sample. The Republic of Türkiye is shown among countries that are culturally highly intolerant of uncertainty (Hofstede, 1980). Parallel to this, Türkiye has a culture where resistance to change is seen intensely (Karatas & Uzun, 2018). Considering the relationship between OCD and intolerance to uncertainty (OCCWG, 2005), BALCI-TV may play a key role in the following studies on the Turkish population. We would like to draw the attention of researchers to the psychological constructs associated with uncertainty and specifically staying in control and losing control.

## Limitations

The data for this study were obtained online via Google Forms. In other words, face-to-face data could not be collected because it was collected during the Coronavirus-19 pandemic period, so this was the first limitation in the data collection process. The second limitation was the data were obtained from a non-clinical population. Participants who voluntarily participated in the research were university students or newly graduated individuals aged 18–28. Most of the participants in the study were women. The small number of male participants in this study was a limitation. One of the prominent aspects of the present study was its contribution to Turkish literature. There were not enough measuring instruments, especially for OCD and OCD-related disorders. Unfortunately, this situation also limited us in terms of test-criterion validity. The scales we preferred in the study reveal the current limitations. Moreover, cross-cultural comparisons could not be made in this adaptation study because BALCI would be the first adaptation study. Another limitation of the study was that it cannot be compared with adaptation processes to other languages or cultures.

## Conclusion

This scale adaptation study will have important contributions. Firstly, BALCI-TV will enable the beliefs about losing control to be measured in Turkish culture. Thus, this measurement instrument will expand the obsessive–compulsive disorder literature in Turkey. Moreover, the instrument will enable researchers working and conducting research on obsessive–compulsive disorder to focus on obsessive–compulsive disorder related different topics. BALCI-TV was shown to be a valid and reliable measurement instrument consisting of 21 items and 3 factors thoughts/behavior/emotions = factor 1, the importance of staying in control = factor 2, body/bodily functions = factor 3). These may have an important function for Turkish academics and mental health practitioners in measuring OCD-based control and beliefs about losing control in university students. BALCI-TV can play a key role, especially considering that there is limited literature and measurement instruments for OCD and OCD-related disorders. Moreover, BALCI-TV may be beneficial for mental health practitioners. Mental health practitioners can benefit from this measurement instrument in OCD-based cases during therapy and psychological help sessions.

Secondly, the participants in the present study group are between the ages of 18–28. In the following studies, validity, and reliability studies for BALCI-TV for different age periods may accelerate. The present study will be a pioneer in adapting the scale to both the clinical population and more different age periods. Validity and reliability studies can be conducted in other Turkish adult groups who are not university students or in clinical populations diagnosed with OCD in future studies. To the best of our knowledge, no further adaptation studies have emerged for BALCI. This scale adaptation study will provide a comparison opportunity in adapting BALCI to other cultures.

Lastly, considering the relationship between OCD and intolerance to uncertainty, we think that BALCI-TV can be used effectively in studies to be conducted on this subject in the Turkish sample. Herewith, we believe that BALCI-TV which deals with emotions, thoughts, behaviors, and beliefs about control and bodily functions together for clinical and non-clinical populations, can be used to examine OCD and related structures with a comprehensive approach.

## Data Availability

The datasets generated during and/or analyzed during the current study are available from the corresponding author on reasonable request.

## Appendix

Kontrolü Kaybetmeye İlişkin İnançlar Envanteri.^1^

Kontrolü kaybetmeye ilişkin inançlar envanteri, duygu ve düşünceleriniz üzerindeki kontrol etmeye yönelik inançlarınızı ölçmeyi amaçlamaktadır. Bu kapsamda envanterde 21 madde yer almaktadır. Sizden istenen, tüm maddeleri aşağıda yer alan derecelendirmeyi kullanarak (0 = Hiç, 4 = Çok Fazla) kendinize en uygun olan şekilde yanıtlamanızdır.
